# New *ABL1* Kinase Domain Mutations in *BCR::ABL1*‐Positive Acute Lymphoblastic Leukemia

**DOI:** 10.1002/cam4.70317

**Published:** 2024-10-23

**Authors:** Zixuan Li, Danyue Peng, Jun Deng, Lv Xiong, Ping Yin, Jing Hu, Chenjing Qian, Lan Yao, Hua Yin, Mei Hong, Qiuling Wu

**Affiliations:** ^1^ Institute of Hematology, Union Hospital, Tongji Medical College, Huazhong University of Science and Technology Wuhan China; ^2^ Department of Epidemiology and Biostatistics, School of Public Health Tongji Medical College, Huazhong University of Science and Technology Wuhan China; ^3^ Operations Management Department, Union Hospital Tongji Medical College, Huazhong University of Science and Technology Wuhan China; ^4^ Institute of Hematology, the Fifth Medical Center of PLA General Hospital Beijing China; ^5^ Collaborative Innovation Center of Hematology Soochow University Suzhou China

**Keywords:** *ABL1*, acute lymphoblastic leukemia, mutation, tyrosine kinase inhibitors

## Abstract

**Background:**

Since the development of the first‐generation Tyrosine Kinase Inhibitor (TKI), it has played a crucial role in the treatment of *BCR::ABL1*‐positive acute lymphoblastic leukemia (ALL) and chronic myeloid leukemia (CML). However, *ABL1* kinase domain (*ABL1* KD) mutations confer resistance to several TKIs. These mutations have been extensively studied in chronic myeloid leukemia (CML) but less so in *BCR::ABL1*‐positive acute lymphoblastic leukemia (ALL).

**Methods:**

Our study aimed to analyze the the *ABL1* KD mutations in 97 consecutive newly‐diagnosed adults with *BCR::ABL1*‐positive ALL before therapy, in cytogenetic complete remission and at relapse with next generation sequencing (NGS). The relationship between *ABL1* KD mutations and TKI selection was also analyzed.

**Results:**

Previously unreported *ABL1* KD mutations R239G, F401V/L, R516L and K262T were the most prevalent in pre‐therapy and cytogenetic remission samples, whereas T315I/P and P‐loop mutations were most prevalent in relapse samples. R239G, F401V/L, R516L and K262T are related to the *BCR::ABL1* structure, whereas T315I/P and P‐loop mutations directly alter kinase activity. BaF3 cells transfected with *ABL1* KD F401V, K262T, R239G, or R516L mutations were resistant to imatinib but strongly inhibited by olverembatinib with IC50 values of 0.73 to 1.52nM. Meanwhile, olverembatinib had advantages in increasing complete molecular response (CMR) and good prognosis.

**Conclusion:**

Overall, our findings indicate the prevalence and impact of new *ABL1* KD mutations in *BCR::ABL1*‐positive ALL patients, highlighting the necessity for effective therapies targetingthese mutations.

## Graphical abstract

We reported the insight of ABL1 KD mutations, especially the new ones (R239G, F401V/L, R516L, and K262T) located in the area that have not been well explored yet. Clinical observation and cell viability detection demonstrated that these new mutations possessed resistance to imatinib but remained sensitive to olverembatinib.

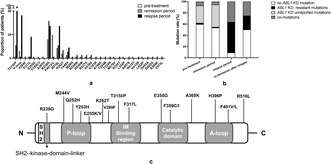

## 1 Introduction

More than 70 mutations were reported in the *BCR::ABL1* kinase domain (KD) in people with *BCR::ABL1*‐associated leukemias. These mutations occur predominantly at four sites: (1) ATP binding P‐loop, (2) imatinib binding region, (3) catalytic domain, and (4) activation (A)‐loop [1]. These mutations are extensively studied in chronic myeloid leukemia (CML), compromise tyrosine kinase inhibitor (TKI) binding sterically, elimination of direct contacts and/or favoring an active conformation of the *ABL1* KD [2, 3]. However, *ABL1* KD mutations are relatively unstudied in *BCR::ABL1*‐positive acute lymphoblastic leukemia (ALL) [4, 5]. We interrogated samples with *BCR::ABL1*‐positive ALL in several leukemia stages for *ABL1* KD mutations using next generation sequencing (NGS). We identified several previously unreported mutations and correlations with response to TKIs.

## 2 Materials and Methods

### 2.1 Subjects and Therapy

We totally selected 103 subjects with *BCR::ABL1*‐positive ALL seen at Huazhong University of Science and Technology affiliated Union Hospital 2017–2023, and their clinical details are shown in Table 1. 218 non‐random frozen bone marrow samples retrospectively collected pre‐therapy (*n* = 82), in complete cytogenetic remission (*n* = 114), and at relapse (*n* = 22) in 97 consecutive newly diagnosed adults were tested for *ABL1* KD mutations by NGS. Mutations not reported in the National Center for Biotechnology Information (NCBI), International Cancer Genome Consortium (ICGC), and Ensemble Database were defined as new ones. T315I/P, M244V, Y253H, E255K, F317L, F359C/I, A365K, H396P, Q252H, E355G, and V289F were defined as *ABL1* KD TKI‐resistant mutations based on prior datasets. Co‐mutations were defined as TKI‐resistant mutation based on prior datasets along with new *ABL1* KD mutations in a subject in a disease stage.

**TABLE 1:** Patient demographics and clinical characteristics.

	*BCR::ABL1*‐positive ALL
Totals pts, *n*	103
Males/females	52/51
Median age at diagnosis, years	39.64
Marrow blast	74.45%
Initial BCR ABL IS	67.41%
WBC count, G/L	53.34
PLT count, G/L	87.20

TKI regimens of 92 subjects were collected, and TKI selection was according to the physician and patient choice. The first generation (1st G) TKI clinically used in our research was imatinib. The second generation (2nd G) TKI included dasatinib and flumatinib. The third generation (3rd G) TKI mostly referred to olverembatinib, and seldom patients chose ponatinib. Meanwhile, those subjects were received combined vincristine‐prednisone (VP)‐based induction regimen and hyper‐fractionated cyclophosphamide, vincristine, doxorubicin, and dexamethasone (Hyper‐CVAD) consolidation regimen.

### 2.2 NGS

NGS of the *ABL1* KD used a panel of 6300 bp amplicons generated by reverse transcription multiplex polymerase chain reaction of total RNA, which was extracted from subjects' bone marrow using TriZol reagent (Invitrogen, Carlsbad, CA, USA) according to the manufacturer's protocol. RNA was converted to cDNA with the HiScript III RT SuperMix for qPCR (+gDNA wiper) kit (Cat. R323‐01, Vazyme, Nanjing, China). Amplification was done using the MultipSeq Library Prep Kit (Cat. M61022, iGeneTech, Beijing, China). Adapters and indexes were introduced in the target amplicons using the MultipSeq CDI Primer (5 μM each, for Illumina, plate) (Cat. M70062, iGeneTech, Beijing, China) during the 2nd amplification round. Target amplicons were purified using the Hieff NGS DNA Selection Beads (Cat. 12601ES56, Yeasen, Shanghai, China). A dsDNA HS Assay Kit for Qubit (Cat. 12640ES76, Yeasen, Shanghai, China) was used to quantify concentrations of the resulting sequencing libraries. Paired‐end sequencing was done using an Illumina system following Illumina‐provided protocols for 150 bp paired‐end sequencing. NGS data was mapped to the human reference sequence hg19 (GRCh37) using the SAMtools software and annotated nucleotide variants in *ABL1* KD according to the GenBank No. NM_005157.5. ANNOVAR software was used to annotate the nucleotide variants in the ABL1 kinase region [1]. Quality of NGS data was assessed using Fastp software [6]. Sequencing quality of each sample was assessed using quality indicators: (1) total reads ≥ 700,000; (2) total reads specificity ≥ 75%; and (3) sequencing depth ≥ 500 00 × for the targeted designed *BCR:ABL1* KD. We set up negative control at 10% of the total number of samples. Absence of mutations detected in the negative control represented a passing test for samples from the same batch.

### 2.3 Cell Culture and Electroporation Transduction

BaF3 cells (KYinno Biotechnology, Beijing, China) were cultured with RPMI 1640 Medium (#C11875, Gibco, NY, USA) plus 10% fetal bovine serum (FBS) (#10099‐141C, Gibco) and 1% penicillin/streptomycin (P/S) (#15140–122, Gibco) in a 37°C incubator with humidified 5% CO_2_. BaF3 cells were harvested and re‐suspended at 1 × 10 [7] cells/mL with pre‐chilled 1640 medium (C11875, Gibco). Cells were transduced with different vector plasmids (*BCR::ABL1* F401V, *BCR::ABL1* K262T, *BCR::ABL1* R239G, and *BCR::ABL1* R516L) and control plasmid (*BCR::ABL1* WT) (GENEWIZ, Suzhou, China) by electrotransfer. 400 μL of the cell suspension was placed in a precooled 0.4 cm Gene MicroPulser Electroporation Cuvette (#1652081, BIO‐RAD, Dryden Vale, UK) and mixed with 5 ug of plasmid. Cuvettes were placed into an electroporator (Model 1652100, Dryden Vale, BIO‐RAD), and a square pulse (260 V/950 uF) was generated, after which the cell suspension in the cuvettes was aspirated and transferred to a 6‐well plate containing prepared RMPI 1640 medium containing 20% FBS and 10 ng/mL IL‐3 (Z03111, GenScript, Oxford, UK). Cells were centrifuged the next day, and the medium was replaced with RMPI 1640 medium containing 10% FBS (IL‐3 free).

### 2.4 Drug Treatment and Cell Viability Detection

*BCR::ABL1* F401V, *BCR::ABL1* K262T, *BCR::ABL1* R239G, *BCR::ABL1* R516L, or *BCR::ABL1* WT transduced BaF3 cells were treated with nine serial concentrations of imatinib (0.18–4.0 mol/L) or dasatinib, flumatinib, or olverembatinib (0.0152–100.00 μmol/L) for 72 h in a CO_2_ incubator at 37°C. Experiments were done in triplicate. At the end of culture, cell viability was assayed using a CellTiter‐Glo (CTG) Luminescent Cell Viability Assay Kit (G7558, Cilomab, London, UK) based on measuring cellular adenosine triphosphate (ATP) levels using the manufacturer's instructions. Equal volumes of reagents were added to each test well after equilibration at 24°C, and chemiluminescence was quantitated using a luminometer (H1MF, Bio‐Tek, Vermont, USA). The sigmoidal dose–response curves (variable slope) and IC_50_ values were obtained using GraphPad prism Software [7].

#### 2.4.1 Polymorphism Phenotyping V2 (PolyPhen‐2)

The Bioinformatics tools like PolyPhen‐2 (http://genetics.bwh.harvard.edu/pph2/) online web servers were used to predict the impact of the stability and function of novel mutations identified on the *BCR::ABL1* protein [8]. “PolyPhen‐2 score” is the probability of the substitution being damaging, and a higher score means single‐nucleotide variants (SNVs) are more deleterious; “sensitivity” and “specificity” correspond to prediction confidence. The predicted damaging effect is also indicated by a vertical black marker inside a color gradient bar, where green is benign and red is damaging [9].

### 2.5 Statistics

Fisher's exact test was employed to test categorical differences, and Wilcoxon signed‐rank test was employed to check differences of measurable variation. The exact logistic regression model was adopted for prognosis analysis. We selected *ABL1* KD mutations as independent variables and relapse as the dependent variable in the exact logistic regression model [10, 11]. The significance cutoff was set as *p* < 0.05. Analysis was done using the software SAS (Statistical Analysis System) [12].

## 3 Results

### 3.1 Distribution of *ABL1* KD Mutations

**FIGURE 1:**
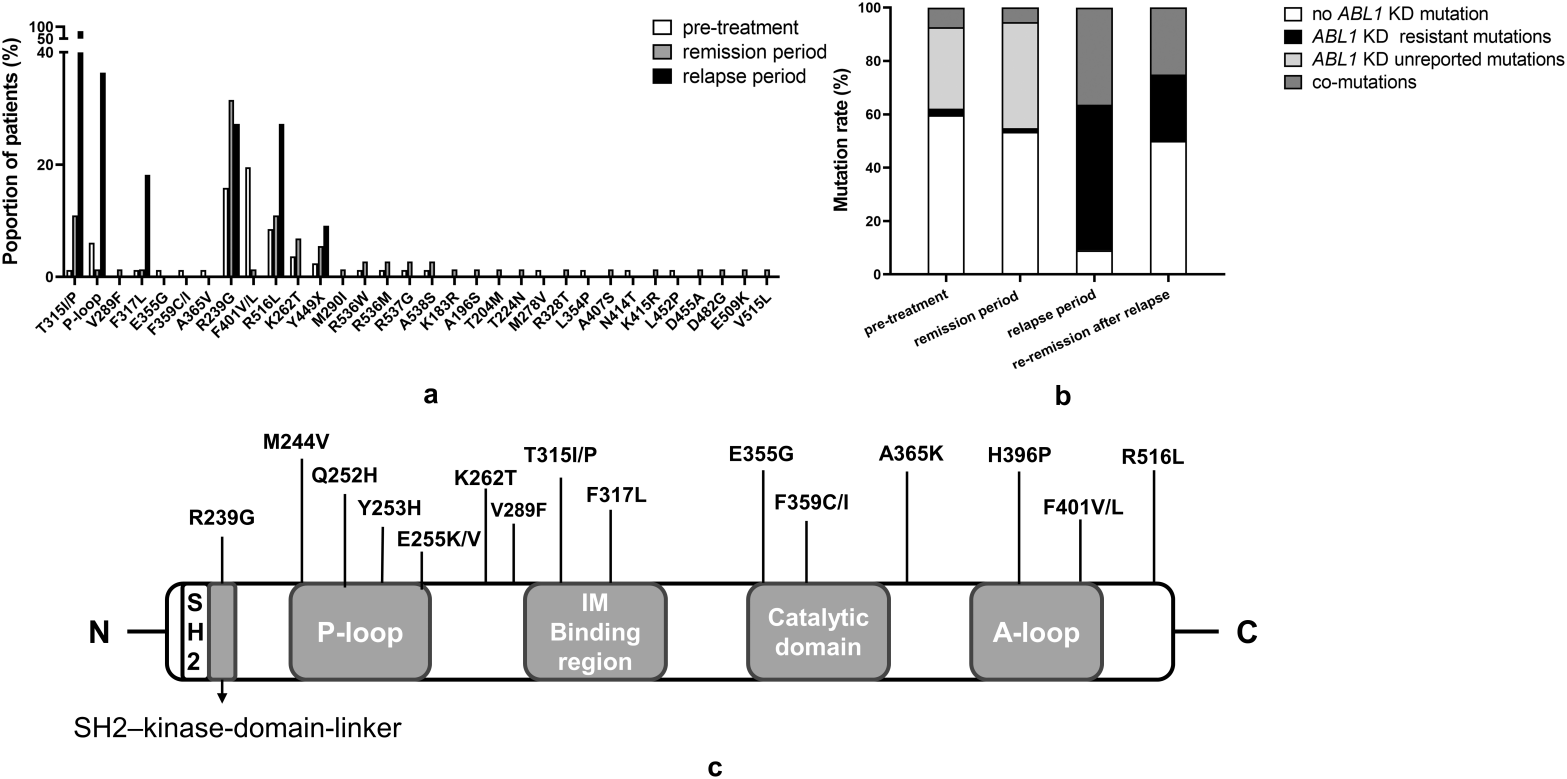
The characteristic of *ABL1* KD mutations in *BCR::ABL1‐*positive ALL detected by NGS. (a) Relative proportions of ABL1 KD mutations found in *BCR::ABL1‐*positive ALL subjects at three disease stages (pre‐treatment, remission, and relapse). (b) Dynamic change of *ABL1* KD mutation types during three disease stages in *BCR::ABL1*‐positive ALL subjects. (c) ABL1 KD mutations detected in this study are demonstrated according to the sites of mutations.

*ABL1* KD mutations are listed in Table S1. We detected 38 *ABL1* KD mutations, 26 of which were new and 12 of which were TKI‐resistant based on reports in prior datasets. R239G, F401V/L, R516L, and K262T were the most common among new mutations, whereas T315I/P and P‐loop mutations including M244V, Q252H, Y253H, and E255K/V were the most frequent resistant mutations (Figure 1a,c). The other new *ABL1* KD mutations appeared to be random and incidental, forming clusters and not consistently detected in subsequent stages.

Seven resistant and 14 new mutations were detected in 82 *BCR::ABL1‐*positive ALL subjects pre‐therapy, and P‐loop mutations were most prevalent (*n* = 5) among resistant mutations. T315I (*n* = 1) and other resistant mutations were uncommon. F401V/L (*n* = 16), R239G (*n* = 13), R516L (*n* = 7), and K262T (*n* = 3) were dominant new mutations detected. Twenty‐five pretherapy samples had new mutations, 2 had resistant mutations, and 6 had co‐mutations.

Four samples had low level T315I mutations in cytogenetic complete remission. Variant allele frequency (VAF) of T315I increased in their relapse samples. Thirty‐three subjects had new mutations, including R239G (*n* = 23), R516L (*n* = 7), and K262T (*n* = 4) in cytogenetic complete remission. We detected 13 resistant mutations with T315I predominating (*n* = 9), followed by P‐loop mutations (*n* = 4) in 11 relapse samples. New mutations detected in relapse samples were R239G (*n* = 3), R516L (*n* = 3), and Y449X (*n* = 1; Figure 1A).

We grouped *ABL1* KD mutations as no mutation, resistant mutations, new mutations, and co‐mutations in subjects to further verify the distribution of *ABL1* KD mutations according to disease stages (Figure 1B). New mutations were mostly found in subjects untreated or in remission, while resistant mutations increased in relapsed ones. There was statistically significant difference in the distribution of *ABL1* KD mutation types at three sampling intervals (*p* < 0.0001). The re‐remission group was excluded because of the limited number (*n* = 4).

### 3.2 PolyPhen‐2 Score Calculation

**FIGURE 2:**
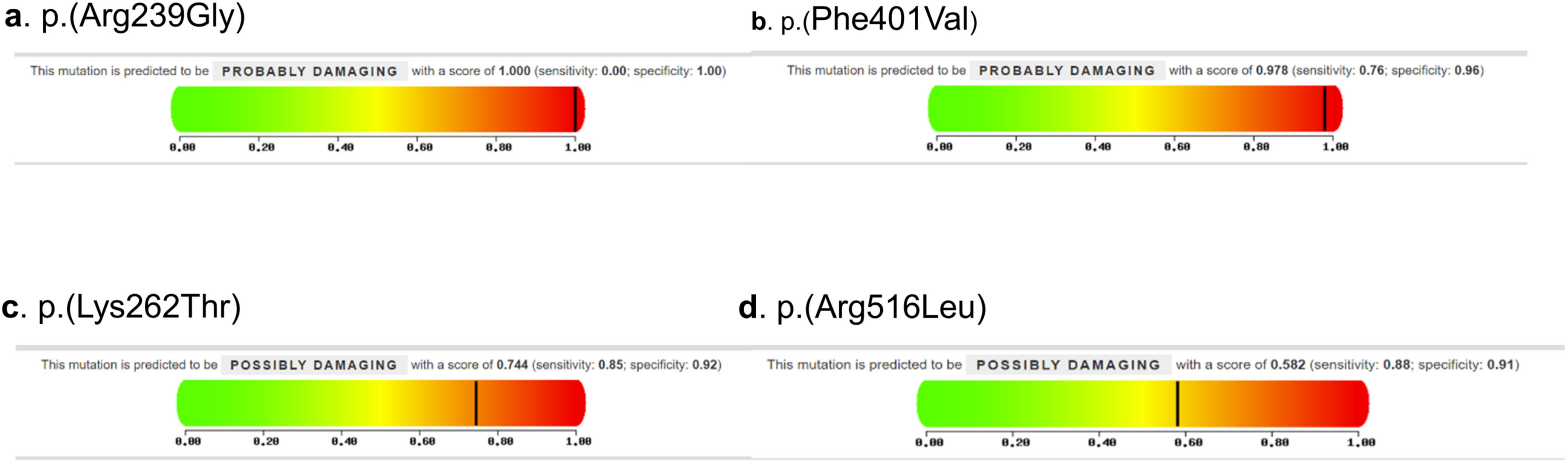
The analysis of mutant *BCR::ABL1* sequences when submitted to the PolyPhen‐2 online server (a–d).

Next, we used PolyPhen‐2 to estimate the possible impact of amino acid substitution on the structure and function of *ABL*1 KD mutations (Table S2). R239G, F401V, K262T, and R516L are predicted to damage the *ABL1* structure, with their PolyPhen‐2 score > 0.5, and the results are shown in Figure 2. R239G and F401V have PolyPhen‐2 scores of 1.000 and 0.978, which indicates that their SNVs possibly damage *BCR::ABL1* KD. K262T and R516L had scores of 0.744 and 0.582, indicating possibly damage to the protein [8, 13].

### 3.3 The Correlation Between Different *ABL1* KD Mutations and Clinical Characteristics at the Pre‐Therapy Stage

To investigate whether mutations detected at newly diagnosed cases were associated with adverse clinical characteristics, a number of baseline clinical characteristics (gender, age, white blood cell [WBC] count, platelet [PLT] count, bone marrow, *BCR::ABL1* International Standard Value [IS], and declined *BCR::ABL1* IS percent after the first course) were analyzed with different mutation status in pre‐treatment subjects by Wilcoxon signed‐rank test analysis in 82 pre‐treatment patients.

Age in the co‐mutated group was the highest (50.67 years), followed by the new mutation group (37.76 years) or un‐mutated group (38.06 years) (*p* = 0.0676, *p* = 0.0745) (Figure S1a). *BCR::ABL1*‐positive ALL subjects with co‐existing mutation had lower PLT count at diagnosis (33.4 G/L) (Figure S1b). Furthermore, a higher percent of marrow blast was observed in subjects without *ABL1* KD mutation compared with those with resistant or new mutation (83.00% vs. 45.20% or 67.27%) (*p* = 0.0503, *p* = 0.0197). Subjects with co‐existing mutation were observed with high marrow blast (81.25%) and initial *BCR::ABL1 IS* (100.15%) as well (Figure S1d,e). No significant difference was observed in other clinical characteristics.

### 3.4 The Predictive Value of New *ABL1* KD Mutations for Prognosis

Furthermore, we furtherly analyzed the effect of new mutations at the pre‐therapy stage on the prognosis (relapse) by exact logistic regression. Among 16 F401V/L mutated subjects, 9 of them received 1st G TKI, 2 of whom relapsed. One died after chimeric antigen receptor T‐cell (CAR‐T) therapy and one continued to follow‐up after switching to 2nd G TKI therapy and hematopoietic stem cell transplantation (HSCT). Seven subjects received 2nd G TKI, and 3 of them relapsed and died. No F401V/L mutated subjects received 3rd G TKI treatment.

Thirteen subjects were R239G carriers at baseline, three, nine, and one of whom received 1st G, 2nd G, and 3rd G TKI, respectively. All subjects remained in CR, except one patient treated with 2nd G TKI relapsed and died later.

Three subjects were detected with K262T, two of whom received 1st G TKI. One of them was refractory and survived after HSCT. No TKI record was found in the third patient case.

Two, three, and two R516L‐mutated subjects received 1st G, 2nd G, and 3rd G TKI, respectively. One patient who received 1st G TKI relapsed and failed to receive CR after TKI upgrading (Figure S2).

Through exact logistic regression, subjects with F401V/l mutation were associated with possibility of relapse to some extent (*p* = 0.0928) (Table S3).

### 3.5 Clinical Response to Different TKI Treatment in Newly Diagnosed *BCR::ABL1*‐Positive ALL Subjects

**FIGURE 3:**
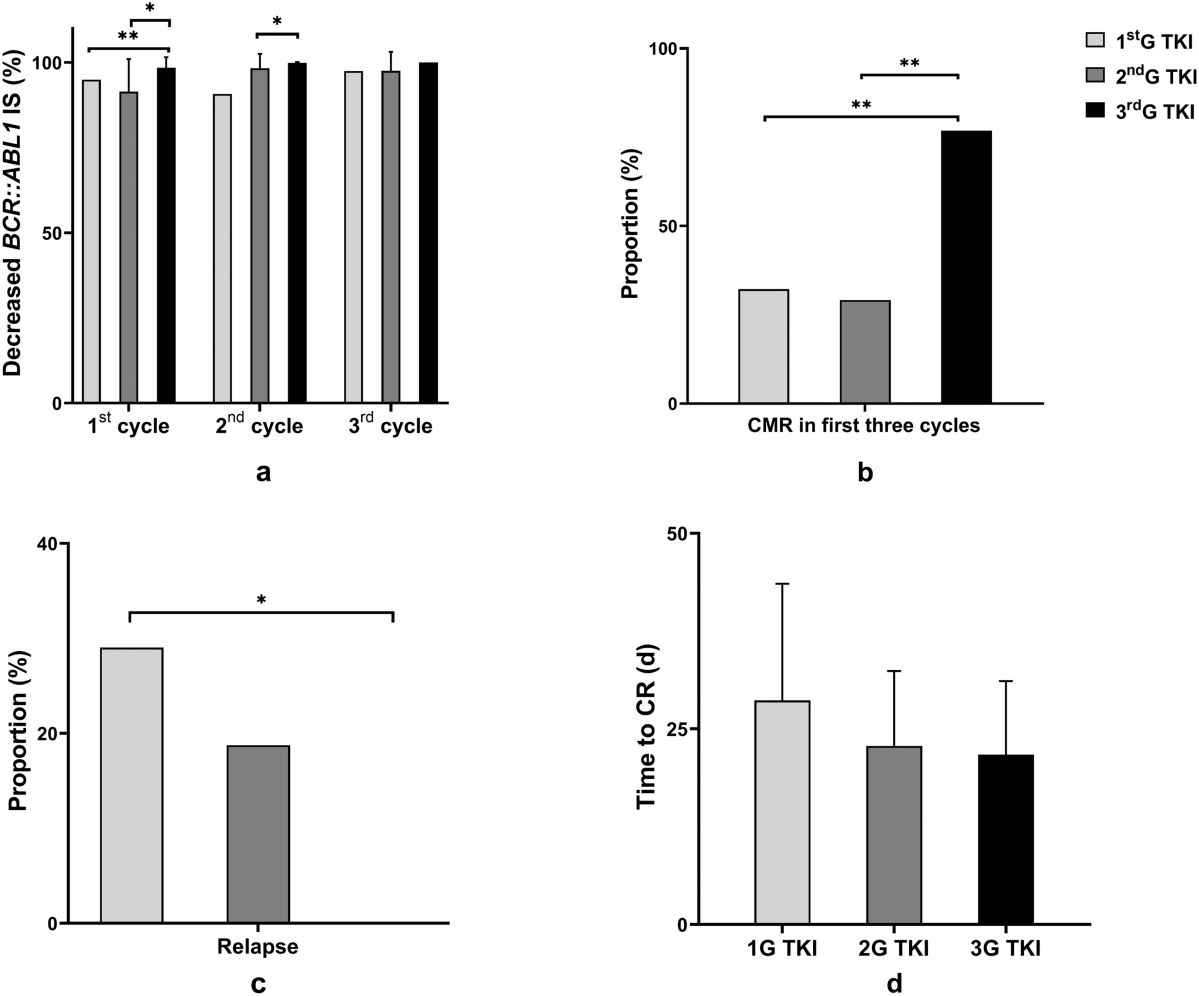
Clinical response in relation to TKI selection in newly diagnosed *BCR::ABL1*‐positive ALL subjects. (a) The ratio of decreased *BCR::ABL1* IS after the 1st, 2nd, and 3rd treatment cycles; (b) proportion of *BCR::ABL1*‐positive ALL patients achieving CMR in first 3 cycles; (c) proportion of *BCR::ABL1*‐positive ALL patients experiencing relapse; (d) median time from TKI treatment to CR. *Note:* *p < 0.05, **p < 0.01.

We retrospectively analyzed the clinical efficacy of three generations of TKIs in 92 *BCR::ABL1*‐positive ALL subjects. The analysis mainly focused on the speed and depth of response and long‐term prognosis. The ratio of decreased *BCR::ABL1 IS* after each treatment cycle compared to the ratio at diagnosis was most pronounced in the subjects treated with 3rdG TKIs. After the first treatment cycle, a significant disparity was observed between 2ndG TKI and 3rdG TKI (*p* = 0.0148), as well as between 1stG TKI and 3rdG TKI (*p* = 0.0085). After the second cycle of treatment, statistical significance was evident in the comparison between 3rdG TKI and 1stG TKI (*p* = 0.0595) and between 3rdG TKI and 2ndG TKI (*p* = 0.0315). Although the difference of the *BCR::ABL1* decrease ratio between 3rdG TKI and 2ndG TKI in the third cycle was not statistically significant (*p* = 0.0857), there was a discernible trend in favor of 3rdG TKI (Figure 3a). In the 3rd G TKI treatment group, the incidence of achieving CMR within the initial three cycles was 76.92% (10/13), which was significantly higher compared to 1stG TKI group (32.26%, 10/31, *p* = 0.0066) and the 2ndG TKI group (29.16%, 14/48, *p* = 0.0010) (Figure 3b). Furthermore, in the 3rdG TKI treatment group, none of the subjects experienced a relapse, while 29.03% subjects in the 1stG TKI (9/13, *p* = 0.0405) and 18.75% in the 2ndG TKI (8/48) eventually relapsed (Figure 3c). Additionally, the average number of days from the initiation of TKI treatment to achieving cytogenetic complete remission was shortest in the 3rd G TKI (21.70 days) compared to the 1stG TKI (28.64 days) and the 2ndG TKI (22.83 days) (Figure 3d).

### 3.6 Impact of TKI Treatment on the Change of *ABL1* KD Mutation

**FIGURE 4:**
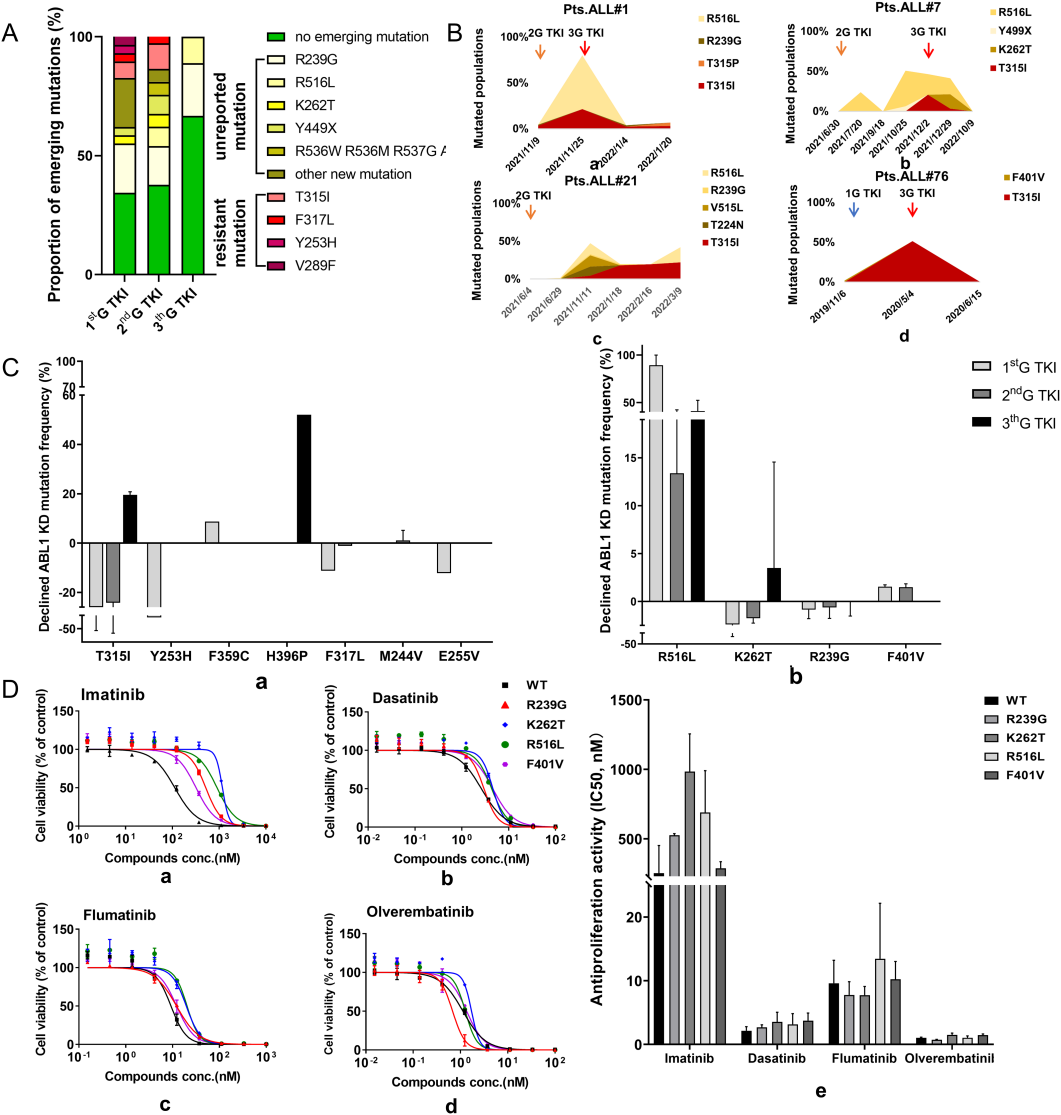
Impact of *ABL1* KD mutations on TKI selection (A) Proportion of *ABL1* KD emerging mutations after TKI exposure; (B) mutated populations rise and fall over time in relation to therapeutic intervention in four subjects; (C) average frequency of *ABL1* KD mutation change exposure to TKI. Proportion declined (positive) or increased (negative) of (a) *ABL1* KD‐resistant mutations and (b) new *ABL1* KD mutations frequency in all patients treated with three different generations of TKI as first‐line or salvage therapy. (D) Effect of TKIs on activity and growth of cells expressing new *ABL1* KD mutations. (a)~(d) BaF3 cells expressing new *ABL1* KD mutations genes were cultured in the presence of imatinib, dasatinib, flumatinib, or olverembatinib for 72 h. (e) The average IC_50_ value of imatinib, dasatinib, flumatinib, or olverembatinib in transfected cell lines after two experiments in vitro.

To elucidate the differential impact of three generations of TKIs on the change of *ABL1* KD mutations, we analyzed the competing populations of mutations affected by TKIs. No *ABL1* KD‐resistant mutation emerged after TKI treatment in all nine *BCR::ABL1*‐positive ALL who initially received 3rdG TKI exposure. In contrast, 17.21% mutations (5/29) in *BCR::ABL1*‐positive ALL subjects emerging after exposure to 1stG TKI were resistant, and 13.51% mutations (5/37) emerged after exposure to 2nd G TKI. Furthermore, compared with 1stG/2ndG TKI, subjects treated with 3rdG TKIs resulted in the highest possibility of not developing any mutations and the lowest possibility of emerging new mutations (Figure 4A). To observe the clinical effect of 3rdG TKI more intuitively, we observed some representative relapsed examples who initially received 1st G/2nd G TKI and switched to 3rdG TKI, as illustrated in Figure 4B; the switch to 3rdG TKI at relapse reduces the mutation frequency. In conclusion, 3rdG TKI was highly effective in inhibiting some *ABL1* KD mutations.

Moreover, we calculated the average frequency change of different *ABL1* KD mutations after treatment with three different generations of TKI as first‐line therapy. Among resistance mutations, T315I was dramatically inhibited by 3rdG TKI, while 1stG/2ndG failed to have an effect on it. H396P was significantly sensitive to 3rdG TKI as well. Y253H, F317L, and E255V expanded after the treatment of 1stG TKI, while F317L was eliminated after 2ndG TKI treatment (Figure 4C,a). Among most frequent new mutations including R239G, F401V, K262T, and R516L, R516L had a higher inhibition treated by 1stG/3rdG TKI compared with 2ndG TKI, and K262T got a better inhibition when treated with 3rdG TKI than 1stG/2ndG TKI (Figure 4C,b).

### 3.7 Testing of New Mutation In Vitro for TKI Resistance

We transfected *BCR::ABL1*‐positive ALL BaF3 cell lines with the plasmid with *BCR::ABL1* wild‐type or *BCR::ABL1* with F401V, K262T, R239G, and R516L mutation genes by electro‐transfection and measured cell viability after exposure to increasing concentrations of imatinib, dasatinib, flumatinib, or olverembatinib. We found that K262T was the most resistant to imatinib with a 4‐fold increase in IC_50_ compared with wild‐type (984.0 nM v.s. 253.3 nM), followed by R516L (689.9 nM), R239G (526.55 nM), and F401V (288.3 nM) (Figure 4D). Sensitivity of these mutants to dasatinib, flumatinib, and olverembatinib was similar to wild‐type. Olverembatinib had the greatest inhibition, with IC_50_ values ranging between 0.73 and 1.52 nM (Figure 4D).

## 4 Discussion

To date, although NGS has been widely used to detect *ABL1* KD mutations in BCR::ABL1‐positive ALL patients, few studies have focused on the clinical characteristics and TKI susceptibility of mutations other than *ABL1* KD‐resistant ones [14, 15, 16, 17]. Therefore, our study mainly focused on the distribution of *ABL1* KD mutations in this population using NGS, emphasizing new mutations and exploring their occurrence regularity and resistance profiles.

We identified 26 new *ABL1* KD mutations in *BCR::ABL*‐positive ALL. Most of these mutations were detected in pretherapy samples and samples from subjects in cytogenetic complete remission. We eventually focused on F401V/L, K262T, R239G, and R516L, which were predicted to have an impact response to TKI‐therapy based on data from Polyphen‐2 prediction and in vitro experiments, indicating these mutations altering the conformation, activity, and binding affinity of the *ABL1* KD.

In our study, new *ABL1* KD mutation F401V/L was predominantly detected in *de novo BCR::ABL1*‐positive ALL subjects, with a potential damage to the *ABL1* structure. F401 is situated within the A‐loop between the N‐lobe and the C‐lobe, transferring between the active state, which provides a platform for substrate binding, and the inactive form, where the activation loop folds back into the ATP binding site to block Mg^2+^ from initiating catalysis. Two states influence binding specificity of several TKIs [18, 19]. A similar mutation F382V, located in the A‐loop, can significantly affect *BCR::ABL1* function and reduce its binding affinity for nilotinib and ponatinib through inducing a conformational change in the Ala‐Pro‐Glu (DFG) motif from its favored “in” state to the “out” state in *ABL1* and thus impairing the catalytic activity of *ABL1* > 20‐fold [3, 18, 20, 21]. The DFG Out conformation has been found to be essential for achieving high‐affinity imatinib binding [22]. This elucidates why BaF3 cells carrying *BCR::ABL1* F401V mutations exhibited comparable sensitivity to imatinib as those with wild‐type counterparts. According to clinical data, however, no relapsed patients were detected with F401V/L, which might be caused by limited relapse subjects. More clinical data is needed to refine the perspective.

The R239G mutation occurs at a significantly higher frequency compared with other novel mutations and can manifest across all stages, particularly prominent during the remission stage in *BCR::ABL1*‐Positive ALL subjects. According to PolyPhen‐2, it was predicted to extremely destroy *BCR::ABL1* and the R239G‐mutated BaF3 cell lines showed resistance to imatinib, which might be attributed to its special location. R239 is inside the SH2–kinase‐domain‐linker, in which mutated residues lead to abnormally phosphorylation and overexpression of *ABL1* with dysregulated Src kinase activity [23, 24]. Olverembatinib has been found to decrease the phosphorylation of Src kinase in Pre‐B cell lines [25], which might explain high sensitivity of olverembatinib to R239G. Similarly, T212R, also located in SH2–kinase‐domain‐linker, is associated with TKI‐resistance in vitro through the stabilization of a more active, but less imatinib accessible conformation [26].

The K262T mutation was exclusively observed in the pre‐treatment and remission stages in *BCR::ABL1*‐Positive ALL subjects. In in vitro experiments, it showed the highest resistance to imatinib, with IC_50_ values increasing fourfold compared to the WT. This resistance is likely due to its proximity to the P‐loop [27, 28]. P‐loop is the most frequently mutated‐occurring domain and directly affects ligand binding in the active site [21, 29]. Mutations like K262T, occurring with the highest frequency in the P‐loop, confer resistance to imatinib [30]. The resistance might be attributed to these mutations destabilizing the particular kinked conformation of the P‐loop that *ABL1* KD adopts when bound to imatinib and destroying the contact and electro‐static interaction between *BCR::ABL1* with imatinib [30, 31].

In our study, R516L ranks as the second most frequent novel mutation after R239G. It demonstrates a threefold increase in IC_50_ of Imatinib compared to the WT, which is possibly attributed to R516's location at the C‐terminal lobe of *ABL1*. Mutations in this region could potentially disrupt the autoinhibited state of *BCR::ABL1*, leading to loss of its inhibitory function [32]. R516 is inside a deep pocket formed by hydrophobic side chains emanating from helices αE, αF, αH, and the new helix, αI. The restructuring of the C‐terminal helix αI is the basis of internal docking of the SH2 and SH3 domain, which is distal to the active site [32, 33].

Many studies have identified additional novel ABL1 KD mutations, for instance C246T, C399T, Q300R, G251E, and N368S [27, 34, 35, 36]. However, no consensus has been reached on the values on these mutations, which may be related to the following three reasons: (1) the relatively small sample sizes; (2) the heterogeneity of the research objects, which included patients with *BCR::ABL1*‐Positive ALL or a preceding antecedent of CML; and (3) limited by assay no new *ABL1* KD mutations detected [37]. Thus far, our study is the largest monocentric study reported aiming to systematically evaluate the characteristics of new *ABL1* KD mutations in adult patients with *BCR::ABL1*‐Positive ALL. However, there is still some limitation in our study since SNVs detected by NGS were called from RNA. SNV differences between DNA–RNA pairs ranged from 10% to 20% [38], which can result in false mutations detected through RNA sequencing. Therefore, subsequent validation of those new mutation detection at the DNA level should be added.

In this cohort study, we also compared the efficacy of TKIs and found that 3^rd^ G TKIs Olverembatinib displayed superior outcomes in terms of reducing the *BCR::ABL1 IS* level, achieving complete molecular response (CMR) within three treatment courses, preventing relapse, and shortening the time to remission in treatment‐naive subjects. Qian J et al. reported that the incidences of achieving MR^4.5^ were 38.9% (48/126) in CML‐CP and 34.2% (13/38) in CML‐AP on olverembatinib therapy at a median of 3 months [39], while in our study, 76.92% (10/13) *BCR::ABL1‐*positive ALL subjects achieved CMR within first 3 cycles. No subjects relapsed later after initial treatment with 3^rd^ G TKI, while some subjects who switched to 3rd G TKI after relapse still faced the possibility of re‐relapse. For instance, patient ALL#92 recurred with a high frequency of T315I (99.7%,), while CMR was obtained in the first course after upgrading to olverembatinib after recurrence; however, T315I and E255K compound mutation recurred in the second course without olverembatinib discontinuation. Currently, 3rd G TKIs are primarily used as second‐line salvage therapies for subjects experiencing cytogenetic relapse with frontline TKI therapy or those with the T315I mutation [40, 41]. Therefore, using 3rd G TKI as a frontline therapy may offer increased benefits to subjects by reducing the risk of relapse. As for *ABL1* KD new mutations, olverembatinib exhibited the lowest IC_50_ and the strongest inhibition potency [5, 13]. These findings collectively highlight the potential of 3rd G TKIs, especially olverembatinib, as a potential therapy to overcome TKI resistance associated with multiple *ABL1* KD mutations.

In summary, we reported the insight of *ABL1* KD mutations, especially the new ones located in the area that have not been well explored yet. R239G, F401V/L, R516L, and K262T were most frequent and possessed resistance to imatinib. The understanding of these mutations is crucial for developing targeted therapies and improving the management of subjects with resistant forms of leukemia. Many research suggested that 2nd G or more advanced TKI should be considered in such subjects [26, 42]. Further investigations are warranted to explore the new *ABL1* KD mutations and develop appropriate therapies against them.

## Author Contributions

**Zixuan Li:** investigation (equal), writing – original draft (equal). **Danyue Peng:** supervision (equal), writing – review and editing (equal). **Jun Deng:** methodology (equal), supervision (equal). **Lv Xiong:** methodology (equal), software (equal), validation (equal). **Ping Yin:** formal analysis (equal), methodology (equal), supervision (equal). **Jing Hu:** formal analysis (equal), methodology (equal), software (equal). **Chenjing Qian:** data curation (equal), resources (equal). **Lan Yao:** data curation (equal), resources (equal). **Hua Yin:** data curation (equal), resources (equal). **Mei Hong:** conceptualization (equal), project administration (equal), supervision (equal). **Qiuling Wu:** project administration (equal), supervision (equal), writing – review and editing (equal).

## Ethics Statement

Protocols were approved by local ethics committees (registration number: [2023]伦审字(0164)号). Informed consent was obtained in accordance with the Declaration of Helsinki.

## Conflicts of Interest

The authors declare no conflicts of interest.

## Supporting information


Data S1.

## Data Availability

For original data, please contact Zixuan Li, 2013724792@qq.com.

## References

[1] S. Soverini , E. Abruzzese , M. Bocchia , et al., “Next‐Generation Sequencing for BCR‐ABL1 Kinase Domain Mutation Testing in Patients With Chronic Myeloid Leukemia: A Position Paper,” Journal of Hematology & Oncology 12, no. 1 (2019): 131, 10.1186/s13045-019-0815-5. 31801582 PMC6894351

[2] T. P. Braun , C. A. Eide , and B. J. Druker , “Response and Resistance to BCR‐ABL1‐Targeted Therapies,” Cancer Cell 37, no. 4 (2020): 530–542, 10.1016/j.ccell.2020.03.006. 32289275 PMC7722523

[3] D. Jones , S. Kamel‐Reid , D. Bahler , et al., “Laboratory Practice Guidelines for Detecting and Reporting BCR‐ABL Drug Resistance Mutations in Chronic Myelogenous Leukemia and Acute Lymphoblastic Leukemia: A Report of the Association for Molecular Pathology,” Journal of Molecular Diagnostics 11, no. 1 (2009): 4–11, 10.2353/jmoldx.2009.080095. PMC2607559 19095773

[4] T. Creasey , E. Barretta , S. L. Ryan , et al., “Genetic and Genomic Analysis of Acute Lymphoblastic Leukemia in Older Adults Reveals a Distinct Profile of Abnormalities: Analysis of 210 Patients From the UKALL14 and UKALL60+ Clinical Trials,” Haematologica 107, no. 9 (2022): 2051–2063, 10.3324/haematol.2021.279177. 34788984 PMC9425332

[5] M. H. Elias , A. A. Baba , H. Azlan , et al., “BCR‐ABL Kinase Domain Mutations, Including 2 Novel Mutations in Imatinib Resistant Malaysian Chronic Myeloid Leukemia Patients‐Frequency and Clinical Outcome,” Leukemia Research 38, no. 4 (2014): 454–459, 10.1016/j.leukres.2013.12.025. 24456693

[6] S. Chen , Y. Zhou , Y. Chen , and J. Gu , “Fastp: An Ultra‐Fast All‐In‐One FASTQ Preprocessor,” Bioinformatics 34, no. 17 (2018): i884–i890, 10.1093/bioinformatics/bty560. 30423086 PMC6129281

[7] K.‐N. Yu , S.‐Y. Kang , S. Hong , and M.‐Y. Lee , “High‐Throughput Metabolism‐Induced Toxicity Assays Demonstrated on a 384‐Pillar Plate,” Archives of Toxicology 92, no. 8 (2018): 2501–2516, 10.1007/s00204-018-2249-1. 29974144

[8] C. Chandrasekhar , P. S. Kumar , and P. Sarma , “Novel Mutations in the Kinase Domain of BCR‐ABL Gene Causing Imatinib Resistance in Chronic Myeloid Leukemia Patients,” Scientific Reports 9, no. 1 (2019): 2412, 10.1038/s41598-019-38672-x. 30787317 PMC6382822

[9] I. Adzhubei , D. M. Jordan , and S. R. Sunyaev , “Predicting Functional Effect of Human Missense Mutations Using PolyPhen‐2,” Current Protocols in Human Genetics 7, no. 20 (2013), 10.1002/0471142905.hg0720s76. PMC4480630 23315928

[10] W. S. Kim , D. Kim , D. W. Kim , et al., “Dynamic Change of T315I BCR‐ABL Kinase Domain Mutation in Korean Chronic Myeloid Leukaemia Patients During Treatment With Abl Tyrosine Kinase Inhibitors,” Hematological Oncology 28, no. 2 (2010): 82–88, 10.1002/hon.918. 19768693

[11] Y. Toyoshima , K. Nemoto , S. Matsumoto , Y. Nakamura , and K. Kiyotani , “SARS‐CoV‐2 Genomic Variations Associated With Mortality Rate of COVID‐19,” Journal of Human Genetics 65, no. 12 (2020): 1075–1082, 10.1038/s10038-020-0808-9. 32699345 PMC7375454

[12] P. Immonen‐Räihä , S. Hätönen , J. Torppa , and A. Toivanen , “A Statistical Analysis System Macro for Age‐Standardized Incidence Rates,” Computer Methods and Programs in Biomedicine 44, no. 2 (1994): 79–83, 10.1016/0169-2607(94)90088-4. 7988119

[13] S. E. Flanagan , A.‐M. Patch , and S. Ellard , “Using SIFT and PolyPhen to Predict Loss‐Of‐Function and Gain‐Of‐Function Mutations,” Genetic Testing and Molecular Biomarkers 14, no. 4 (2010): 533–537, 10.1089/gtmb.2010.0036. 20642364

[14] H. Pfeifer , T. Lange , S. Wystub , et al., “Prevalence and Dynamics of Bcr‐Abl Kinase Domain Mutations During Imatinib Treatment Differ in Patients With Newly Diagnosed and Recurrent Bcr‐Abl Positive Acute Lymphoblastic Leukemia,” Leukemia 26, no. 7 (2012): 1475–1481, 10.1038/leu.2012.5. 22230800

[15] C. Baer , M. Meggendorfer , C. Haferlach , W. Kern , and T. Haferlach , “Detection of ABL1 Kinase Domain Mutations in Therapy‐Naïve BCR‐ABL1‐Positive Acute Lymphoblastic Leukemia,” Haematologica 107, no. 2 (2022): 562–563, 10.3324/haematol.2021.279807. 34758608 PMC8804577

[16] R. Sanchez , S. Dorado , Y. Ruiz‐Heredia , et al., “Detection of Kinase Domain Mutations in BCR::ABL1 Leukemia by Ultra‐Deep Sequencing of Genomic DNA,” Scientific Reports 12, no. 1 (2022): 13057, 10.1038/s41598-022-17271-3. 35906470 PMC9338264

[17] S. Soverini , F. Albano , R. Bassan , et al., “Next‐Generation Sequencing for BCR‐ABL1 Kinase Domain Mutations in Adult Patients With Philadelphia Chromosome‐Positive Acute Lymphoblastic Leukemia: A Position Paper,” Cancer Medicine 9, no. 9 (2020): 2960–2970, 10.1002/cam4.2946. 32154668 PMC7196068

[18] A. A. El Rashedy , P. Appiah‐Kubi , and M. E. S. Soliman , “A Synergistic Combination Against Chronic Myeloid Leukemia: An Intra‐Molecular Mechanism of Communication in BCR‐ABL1 Resistance,” Protein Journal 38, no. 2 (2019): 142–150, 10.1007/s10930-019-09820-z. 30877503

[19] A. Aleksandrov and T. Simonson , “Molecular Dynamics Simulations Show That Conformational Selection Governs the Binding Preferences of Imatinib for Several Tyrosine Kinases,” Journal of Biological Chemistry 285, no. 18 (2010): 13807–13815, 10.1074/jbc.M110.109660. 20200154 PMC2859544

[20] W. W. Chan , S. C. Wise , M. D. Kaufman , et al., “Conformational Control Inhibition of the BCR‐ABL1 Tyrosine Kinase, Including the Gatekeeper T315I Mutant, by the Switch‐Control Inhibitor DCC‐2036,” Cancer Cell 19, no. 4 (2011): 556–568, 10.1016/j.ccr.2011.03.003. 21481795 PMC3077923

[21] T. Xie , T. Saleh , P. Rossi , and C. G. Kalodimos , “Conformational States Dynamically Populated by a Kinase Determine Its Function,” Science 370, no. 6513 (2020): eabc2754, 10.1126/science.abc2754. PMC7920495 33004676

[22] N. M. Levinson , O. Kuchment , K. Shen , et al., “A Src‐Like Inactive Conformation in the Abl Tyrosine Kinase Domain,” PLoS Biology 4, no. 5 (2006): e144, 10.1371/journal.pbio.0040144. 16640460 PMC1450098

[23] O. Hantschel and G. Superti‐Furga , “Regulation of the c‐Abl and Bcr‐Abl Tyrosine Kinases,” Nature Reviews. Molecular Cell Biology 5, no. 1 (2004): 33–44, 10.1038/nrm1280. 14708008

[24] B. B. Brasher and R. A. Van Etten , “C‐Abl Has High Intrinsic Tyrosine Kinase Activity That Is Stimulated by Mutation of the Src Homology 3 Domain and by Autophosphorylation at Two Distinct Regulatory Tyrosines,” Journal of Biological Chemistry 275, no. 45 (2000): 35631–35637, 10.1074/jbc.M005401200. 10964922

[25] W. Ye , Z. Jiang , X. Lu , et al., “GZD824 Suppresses the Growth of Human B Cell Precursor Acute Lymphoblastic Leukemia Cells by Inhibiting the SRC Kinase and PI3K/AKT Pathways,” Oncotarget 8, no. 50 (2017): 87002–87015, 10.18632/oncotarget.10881. 29152059 PMC5675611

[26] D. W. Sherbenou , O. Hantschel , I. Kaupe , et al., “BCR‐ABL SH3‐SH2 Domain Mutations in Chronic Myeloid Leukemia Patients on Imatinib,” Blood 116, no. 17 (2010): 3278–3285, 10.1182/blood-2008-10-183665. 20519627 PMC2995357

[27] S. Soverini , A. Hochhaus , F. E. Nicolini , et al., “BCR‐ABL Kinase Domain Mutation Analysis in Chronic Myeloid Leukemia Patients Treated With Tyrosine Kinase Inhibitors: Recommendations From an Expert Panel on Behalf of European LeukemiaNet,” Blood 118, no. 5 (2011): 1208–1215, 10.1182/blood-2010-12-326405. 21562040

[28] S. Chu , H. Xu , N. P. Shah , et al., “Detection of BCR‐ABL Kinase Mutations in CD34+ Cells From Chronic Myelogenous Leukemia Patients in Complete Cytogenetic Remission on Imatinib Mesylate Treatment,” Blood 105, no. 5 (2005): 2093–2098, 10.1182/blood-2004-03-1114. 15345592

[29] S. Cang and D. Liu , “P‐Loop Mutations and Novel Therapeutic Approaches for Imatinib Failures in Chronic Myeloid Leukemia,” Journal of Hematology & Oncology 1, no. 1 (2008): 15, 10.1186/1756-8722-1-15. 18828913 PMC2567340

[30] M. A. Seeliger , P. Ranjitkar , C. Kasap , et al., “Equally Potent Inhibition of c‐Src and Abl by Compounds That Recognize Inactive Kinase Conformations,” Cancer Research 69, no. 6 (2009): 2384–2392, 10.1158/0008-5472.CAN-08-3953. 19276351 PMC2678021

[31] T. S. Lee , S. J. Potts , H. Kantarjian , J. Cortes , F. Giles , and M. Albitar , “Molecular Basis Explanation for Imatinib Resistance of BCR‐ABL due to T315I and P‐Loop Mutations From Molecular Dynamics Simulations,” Cancer 112, no. 8 (2008): 1744–1753, 10.1002/cncr.23355. 18338744

[32] S. Panjarian , R. E. Iacob , S. Chen , J. R. Engen , and T. E. Smithgall , “Structure and Dynamic Regulation of Abl Kinases,” Journal of Biological Chemistry 288, no. 8 (2013): 5443–5450, 10.1074/jbc.R112.438382. 23316053 PMC3581414

[33] B. Nagar , O. Hantschel , M. A. Young , et al., “Structural Basis for the Autoinhibition of c‐Abl Tyrosine Kinase,” Cell 112, no. 6 (2003): 859–871, 10.1016/s0092-8674(03)00194-6. 12654251

[34] S. Soverini , A. Vitale , A. Poerio , et al., “Philadelphia‐Positive Acute Lymphoblastic Leukemia Patients Already Harbor BCR‐ABL Kinase Domain Mutations at Low Levels at the Time of Diagnosis,” Haematologica 96, no. 4 (2011): 552–557, 10.3324/haematol.2010.034173. 21193419 PMC3069232

[35] W. Wongboonma , W. Thongnoppakhun , and C. U. Auewarakul , “BCR‐ABL Kinase Domain Mutations in Tyrosine Kinase Inhibitors‐Naive and ‐Exposed Southeast Asian Chronic Myeloid Leukemia Patients,” Experimental and Molecular Pathology 92, no. 2 (2012): 259–265, 10.1016/j.yexmp.2012.01.007. 22314255

[36] S. Grammatico , L. Elia , A. L. Peluso , et al., “Increasing the BCR‐ABL Expression Levels and/or the Occurrence of ABL Point Mutations Does Not Always Predict Resistance to Imatinib Mesylate in BCR‐ABL Positive Acute Lymphoblastic Leukemia,” Leukemia Research 33, no. 7 (2009): e73–e74, 10.1016/j.leukres.2008.11.009. 19108887

[37] T. Shi , M. Xie , L. Chen , et al., “Distinct Outcomes, ABL1 Mutation Profile, and Transcriptome Features Between p190 and p210 Transcripts in Adult Philadelphia‐Positive Acute Lymphoblastic Leukemia in the TKI Era,” Experimental Hematology & Oncology 11, no. 1 (2022): 13, 10.1186/s40164-022-00265-2. 35277197 PMC8915539

[38] Y. Guo , S. Zhao , Q. Sheng , D. C. Samuels , and Y. Shyr , “The Discrepancy Among Single Nucleotide Variants Detected by DNA and RNA High Throughput Sequencing Data,” BMC Genomics 18, no. Suppl 6 (2017): 690, 10.1186/s12864-017-4022-x. 28984205 PMC5629567

[39] Q. Jiang , Z. Li , Y. Qin , et al., “Olverembatinib (HQP1351), a Well‐Tolerated and Effective Tyrosine Kinase Inhibitor for Patients With T315I‐Mutated Chronic Myeloid Leukemia: Results of an Open‐Label, Multicenter Phase 1/2 Trial,” Journal of Hematology & Oncology 15, no. 1 (2022): 113, 10.1186/s13045-022-01334-z. 35982483 PMC9389804

[40] H. Qian , D. Gang , X. He , and S. Jiang , “A Review of the Therapeutic Role of the New Third‐Generation TKI Olverembatinib in Chronic Myeloid Leukemia,” Frontiers in Oncology 12 (2022): 1036437, 10.3389/fonc.2022.1036437. 36568202 PMC9772831

[41] E. Jabbour and H. Kantarjian , “Chronic Myeloid Leukemia: 2022 Update on Diagnosis, Therapy, and Monitoring,” American Journal of Hematology 97, no. 9 (2022): 1236–1256, 10.1002/ajh.26642. 35751859

[42] S. Branford , Z. Rudzki , S. Walsh , et al., “Detection of BCR‐ABL Mutations in Patients With CML Treated With Imatinib Is Virtually Always Accompanied by Clinical Resistance, and Mutations in the ATP Phosphate‐Binding Loop (P‐Loop) are Associated With a Poor Prognosis,” Blood 102, no. 1 (2003): 276–283, 10.1182/blood-2002-09-2896. 12623848

