# Supplementary material for: New ABL1 Kinase Domain Mutations in BCR::ABL1‐Positive Acute Lymphoblastic Leukemia
Source: Cancer Med. 2024 Oct 23;13(20):e70317. doi: 10.1002/cam4.70317 (PMC11497109; doi:10.1002/cam4.70317)
Supplement: Supplementary file 1 — Data S1. [file CAM4-13-e70317-s001.zip › Supplemental Materials.docx]

Supplement table 1. All *ABL1* KD mutations detected in *BCR::ABL1*-positive +ALL patients at different disease stages

|  | *BCR::ABL1*-positive +ALL | | | | |  |
| --- | --- | --- | --- | --- | --- | --- |
|  | | Pre-treatment | Remission period 1 | Relapse period | re-remission after relapse | |
| T315I | | 1 | 4 | 9 | 3 | |
| T315P | | 0 | 0 | 0 | 1 | |
| M244V | | 3 | 0 | 1 | 0 | |
| Y253H | | 1 | 0 | 1 | 0 | |
| E255K/V | | 1 | 1 | 2 | 0 | |
| V289F | | 0 | 1 | 0 | 0 | |
| F317L | | 1 | 1 | 2 | 0 | |
| E355G | | 1 | 0 | 0 | 0 | |
| F359C/I | | 1 | 0 | 0 | 0 | |
| A365V | | 1 | 0 | 0 | 0 | |
| R239G | | 13 | 23 | 3 | 0 | |
| R516L | | 7 | 7 | 3 | 1 | |
| K262T | | 3 | 4 | 0 | 1 | |
| Y449X | | 2 | 4 | 1 | 0 | |
| F401V/l | | 16 | 1 | 0 | 0 | |
| R536W | | 1 | 2 | 0 | 0 | |
| R536M | | 1 | 2 | 0 | 0 | |
| R537G | | 1 | 2 | 0 | 0 | |
| A538S | | 1 | 2 | 0 | 0 | |
| V515L | | 0 | 1 | 0 | 0 | |
| M290I | | 0 | 1 | 0 | 0 | |
| T224N | | 0 | 1 | 0 | 0 | |
| A407S | | 0 | 1 | 0 | 0 | |
| M278V | | 1 | 0 | 0 | 0 | |
| L354P | | 1 | 0 | 0 | 0 | |
| N414T | | 1 | 0 | 0 | 0 | |
| D482G | | 1 | 0 | 0 | 0 | |
| L452P | | 1 | 0 | 0 | 0 | |
| K415R | | 0 | 1 | 0 | 0 | |
| T204M | | 0 | 1 | 0 | 0 | |
| K183R | | 0 | 1 | 0 | 0 | |
| E509K | | 0 | 1 | 0 | 0 | |
| A196S | | 0 | 1 | 0 | 0 | |
| R328T | | 0 | 1 | 0 | 0 | |
| D455A | | 0 | 1 | 0 | 0 | |

Supplement table 2. The PolyPhen-2 analysis of new mutants appeared more than once in *BCR::ABL1*.

| Mutation Base  Change | Amino  acid  change | PolyPhen  score | Sensitivity | Specificity |
| --- | --- | --- | --- | --- |
| c.C715G | R239G | 1.000 | 0.00 | 1.00 |
| c.G1607T | R536M | 0.998 | 0.73 | 0.96 |
| c.A1606T | R536W | 0.997 | 0.41 | 0.98 |
| c.T1201G | F401V | 0.978 | 0.76 | 0.96 |
| c.A785C | K262T | 0.744 | 0.85 | 0.92 |
| c.G1547T | R516L | 0.582 | 0.88 | 0.91 |
| c.A1609G | R537G | 0.474 | 0.89 | 0.90 |
| c.G1612T | A538S | 0.077 | 0.93 | 0.85 |

Supplement table 3. Odds ratio (OR) with 95% confidence interval (CI) of new *ABL1* KD mutations and relapse.

| Mutation | OR | 95%CI | *P* |
| --- | --- | --- | --- |
| R239G | 0.241 | 0.004-2.505 | 0.4013 |
| F401V_l | 5.311 | 0.789-36.828 | 0.0928 |
| R516L | 2.011 | 0.035-17.043 | 0.9565 |
| Y449X | 2.038 | 0-24.910 | 0.6908 |
| K262T | 2.847 | 0-28.474 | 0.7594 |
| R536W | 6.708 | 0-127.456 | 0.8703 |
| M278V | 1.499 | 0-28.474 | 0.5998 |


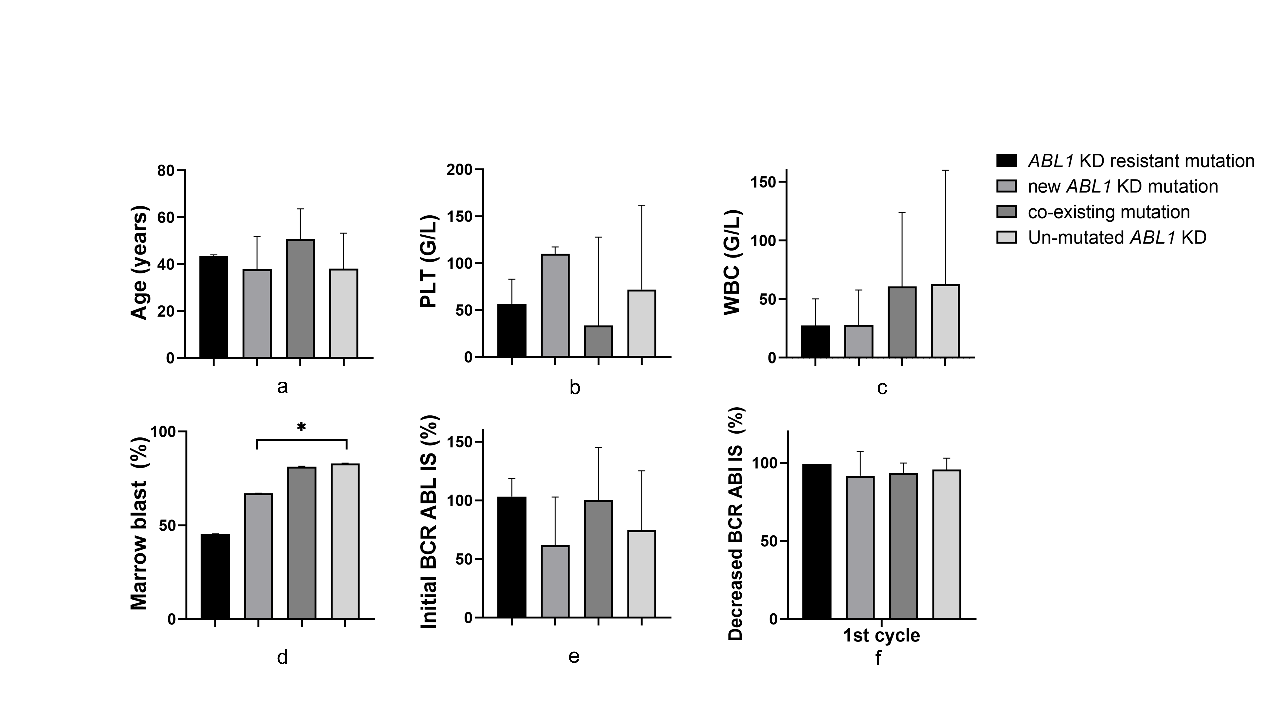


Supplement figure 1. Difference of baseline clinical variables (a. age, b. PLT count, c. WBC count, d. marrow blast, e. initial BCR ABL IS, f. decreased BCR ABL IS after the first cycle) in four mutation status before treatment (single ABL1 KD resistant mutation, single ABL1 KD unreported mutation, resistant and unreported co-existing mutation, and un-mutated ABL1 KD) in *BCR::ABL1*-positive ALL patients.


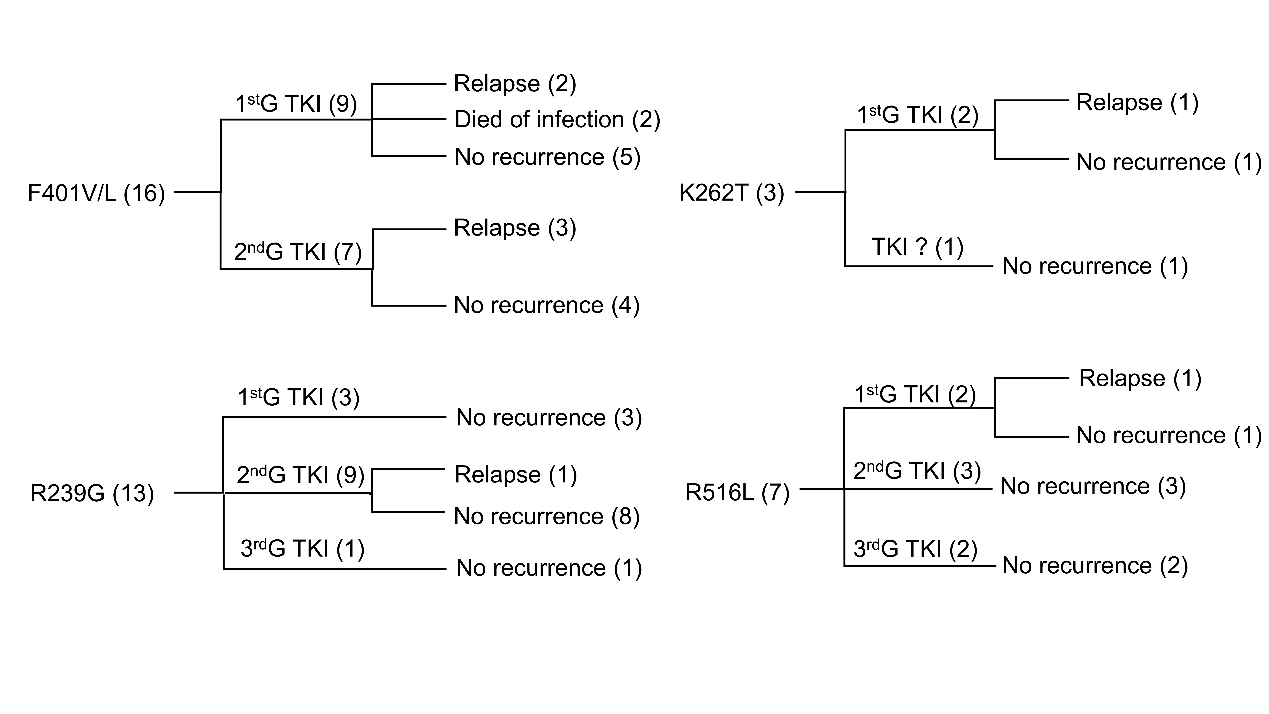


Supplement figure 2. The prognosis for subjects with *ABL1* KD mutation F401V/L, R239G, K262T and R516L at diagnosis.
